# Type 1 Diabetes Mellitus Virtual Patient Network as a Peer Support Community: Protocol for Social Network Analysis and Content Analysis

**DOI:** 10.2196/18714

**Published:** 2020-08-31

**Authors:** Nancy Wu, Anne-Sophie Brazeau, Meranda Nakhla, Deborah Chan, Deborah Da Costa, Geetha Mukerji, Sonia Butalia, Daniele Pacaud, Mélanie Henderson, Constadina Panagiotopoulos, Elham Rahme, Kaberi Dasgupta

**Affiliations:** 1 Centre for Outcomes Research and Evaluation Research Institute of the McGill University Health Centre Montreal, QC Canada; 2 School of Human Nutrition McGill University Montreal, QC Canada; 3 Institute of Health Policy, Management and Evaluation University of Toronto Toronto, ON Canada; 4 Department of Medicine Cumming School of Medicine University of Calgary Calgary, AB Canada; 5 Department of Community Health Sciences Cumming School of Medicine University of Calgary Calgary, AB Canada; 6 Department of Pediatrics Alberta Children's Hospital University of Calgary Calgary, AB Canada; 7 Division of Endocrinology and Diabetes Faculty of Medicine University of Montreal Montreal, QC Canada; 8 Division of Endocrinology Faculty of Medicine University of British Columbia Vancouver, BC Canada; 9 Department of Medicine McGill University Montreal, QC Canada

**Keywords:** type 1 diabetes, youth, social network analysis, content analysis, social media

## Abstract

**Background:**

Type 1 Diabetes Mellitus Virtual Patient Network (T1DM-VPN) is a private Facebook group for youths with type 1 diabetes mellitus (T1DM) in Canada intended to facilitate peer-to-peer support. It was built on the finding that stigma is prevalent among youth with T1DM and impedes self-management.

**Objective:**

We aim to determine if T1DM-VPN provides support as intended and to ascertain what type of members provide support. Specifically, we will (1) identify text consistent with any one of 5 social support categories, (2) describe the network by visualizing its structure and reporting basic engagement statistics, and (3) determine whether being a designated peer leader is related to a member’s centrality (ie, importance in the network) and how frequently they offer social support.

**Methods:**

We will manually extract interaction data from the Facebook group (posts, comments, likes/reactions, seen) generated from June 21, 2017 (addition of first member), to March 1, 2020. Two researchers will independently code posts and comments according to an existing framework of 5 social support categories—informational, emotional, esteem, network, and tangible—with an additional framework for nonsocial support categories. We will calculate how frequently each code is used. We will also report basic engagement statistics (eg, number of posts made per person-month) and generate a visualization of the network. We will identify stable time intervals in the history of T1DM-VPN by modeling monthly membership growth as a Poisson process. Within each interval, each member’s centrality will be calculated and standardized to that of the most central member. We will use a centrality formula that considers both breadth and depth of connections (centrality = 0.8 × total No. of connections + 0.2 × total No. of interactions). Finally, we will construct multivariate linear regression models to assess whether peer leader status predicts member centrality and the frequency of offering social support. Other variables considered for inclusion in the models are gender and age at diagnosis.

**Results:**

T1DM-VPN was launched in June 2017. As of March 1, 2020, it has 196 patient-members. This research protocol received ethics approval from the McGill University Health Centre Research Ethics Board on May 20, 2020. Baseline information about each group member was collected upon addition into the group, and collection of interaction data is ongoing as of May 2020.

**Conclusions:**

This content analysis and social network analysis study of a virtual patient network applies epidemiological methods to account for dynamic growth and activity. The results will allow for an understanding of the topics of importance to youth with T1DM and how a virtual patient network evolves over time. This work is intended to serve as a foundation for future action to help youth improve their experience of living with diabetes.

**International Registered Report Identifier (IRRID):**

PRR1-10.2196/18714

## Introduction

### Background

Type 1 diabetes mellitus (T1DM) is a chronic condition whereby one’s immune system attacks the pancreas, rendering it unable to produce adequate insulin for glucose entry from the circulation and into the cells of the body to fuel metabolism. This differs from the general pathology in type 2 diabetes, which is one of resistance to the action of insulin, often related to low physical activity and excess adiposity. Patients with T1DM administer insulin as a medication, adjusting doses in relationship to food intake and physical activity. They are challenged by a narrow therapeutic window, navigating a tight balance between preventing low glucose levels and high glucose levels. Low levels, or hypoglycemia, can lead to confusion, loss of consciousness, and even death. Persistently high levels over time can damage blood vessels, resulting in blindness, renal injury, cardiovascular disease, stroke, and a multitude of other complications. The visibility of hypoglycemic symptoms, blood glucose testing equipment, and insulin administration, among other tasks and public misconceptions about T1DM, may lead to stigma.

Indeed, in a previous study, we determined that approximately 65% of Canadian youths (ie, aged 14 to 24 years) with T1DM experience stigma and that it is associated with greater probability of both severe hypoglycemia and high average glucose levels, specifically elevated glycated hemoglobin (HbA_1C_) [1]. The Type 1 Diabetes Mellitus Virtual Patient Network (T1DM-VPN) is a private Facebook group launched in 2017 to help facilitate peer-to-peer support for these youths, allow youth to share experiences of living with T1DM, and perhaps mitigate stigma. We now aim to assess whether the Facebook group is providing the support it is intended to provide. We present herein a detailed protocol for this analysis.

We first provide an overview of T1DM management, existing web platforms for patients and their families, and the development of T1DM-VPN towards its current structure.

### Barriers to T1DM Management

In Canada, 24,170 children and adolescents and 84,380 young adults have diabetes [2], over 90% of which is T1DM [3,4]. T1DM management requires insulin pump or injection use, finger pricks for blood glucose testing, and attention to food choices, meal timing, and physical activity levels. However, youth must also manage challenges of identity development, education and career choices, and peer pressure. Managing both sets of needs can be complicated by stigma. Stigma is defined as real or perceived negative social judgement from one’s surroundings or oneself [5]. Our Canada-wide study of 380 youths determined that 65% report some degree of stigma (ie, endorsed one or more of 3 key items on a stigma subscale) [1]. Youths experiencing stigma were twice as likely to have either an HbA_1c_ above 9% or one or more severe hypoglycemic events in the prior year. HbA_1c_ is a measure of the average level of blood sugar over the past 2 to 3 months, and higher levels indicate greater risk of serious diabetes-related complications, such as cardiovascular disease and kidney, eye, and nerve damage [6]. Severe hypoglycemia may also cause distressing conditions such as confusion, loss of consciousness, and even death [7,8]. Our study also determined that stigma was associated with a reduced sense of well-being and less self-efficacy for self-management. Most participants reported that they did not personally know anyone with T1DM and desired social support specifically from peers with T1DM.

### Existing Web Platforms

Web platforms for patients with T1DM vary in reach and in purpose. On Twitter, hashtags are used by people all over the world to connect on specific topics. For example, the #OpenAPS hashtag is used by patients and caregivers to vocalize their experience using do-it-yourself (DIY) innovations that bridge communication between insulin pumps and glucose monitors [9]. Meanwhile, a Facebook group numbering over 27,000 members provides practical aid in using DIY programs [10]. Some caregivers of children with T1DM publish blogs in order to publicly express their experiences and feelings, with additional caregivers commenting [11]. Some youths initiate local university-based diabetes student organizations with a respective social media platform, including in Canadian towns such as Toronto and London. Other platforms are managed professionally. For example, Beyond Type 1 is an organization that amalgamates practical resources and stories on a website, including some specific to Canada. They also have an app where registered adults and teenagers can socialize via public posts and comments. Social media groups may be generated by professionals as well; one clinical team in Australia created a small (34 members) private Facebook group for youths with T1DM as part of a 12-week trial to support their transition to independent self-care [12].

In this landscape, T1DM-VPN is distinguished as a joint initiative between health professional researchers and youths with T1DM. It is funded by Diabetes Canada as well as the Canadian Institutes of Health Research (CIHR) grant, specifically their Strategy for Patient-Oriented Research – Patient-Oriented Research Collaboration Grants. Its core feature is a private Facebook group. Eligible members are Canadian youths (ie, aged 14-24 years) with T1DM. Many T1DM-specific groups have been initiated organically on Facebook. However, none are known to be specific to Canadian youth, who can benefit from region-specific information and from interacting almost instantly with those who face similar everyday challenges across the country. It is open only to patients, not to parents or other caregivers, at the specific request of its founding patient-partners. Its 3 goals are (1) to be a community of support, (2) to identify the issues that matter to patients, and (3) to establish a platform for action and empowerment.

### Development of T1DM-VPN

We collaborated with 2 patient-partners to inform its development, 1 youth with T1DM and 1 adult with T1DM with experience as a certified peer leader for a chronic illness self-management program at the McGill University Health Centre (MUHC) called My Tool Box. My Tool Box is based on the Stanford model of chronic disease self-management, which allows individuals living with a chronic illness to engage in group discussions on self-care led by trained peer leaders who also live with chronic illness [13]. This program was discontinued at the MUHC but our patient-partner’s experience with it was important in the training of our peer leaders.

We recruited youth peer leaders for T1DM-VPN by asking our coinvestigators across Canada to approach any patients who they thought would be a champion for T1DM and by approaching patients who submitted moving testimonies from our original study on stigma and T1DM. They were told that responsibilities include starting conversations in the group and having one-on-one conversations if requested by a member.

At the 2017 Diabetes Canada conference in Edmonton, several peer leaders participated in the satellite workshop that we organized to encourage group cohesion, provide training in peer support, discuss goals, and craft community guidelines. Each peer leader receives a stipend (Can $25, or US $18.88, per month) to support involvement in T1DM-VPN. They take an active role in initiating conversations, sharing information, and answering questions. They provide input on recruitment strategy. They reach out to our team of clinicians and researchers for information.

**Figure 1:**
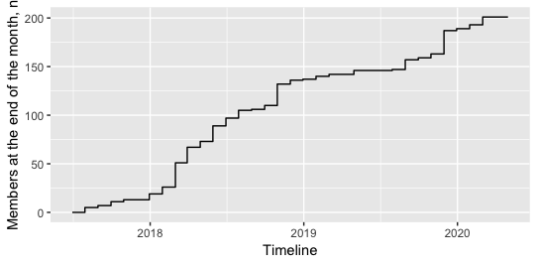
Number of Type 1 Diabetes Mellitus Virtual Patient Network Facebook group members from June 21, 2017, to February 4, 2020.

Some regular T1DM-VPN members are recruited from among participants in our original study on stigma. Regular members are continuously recruited through the clinics of our coinvestigators and via posters displayed in clinics and posted online on diabetes-related Facebook pages and websites. Addition of members began in June 2017 and membership passed 200 on February 4, 2020 (Figure 1). Of these members, 5 are members of our research team, including an administrator account. The rest are youths with T1DM from over 20 towns and cities in all 10 Canadian provinces.

The administrator account is used by the research team to oversee the group. We add eligible members, help answer questions that may require professional input, and ensure that the code of conduct (eg, no disrespectful behavior) is followed. Fortunately, our members have only behaved in a courteous and supportive manner and we have never had to intervene. As patient membership approaches 200, we aim to determine if the project goals are being fulfilled. This will help inform strategies to strengthen and sustain the network.

T1DM-VPN members are aware that the private Facebook group was created by researchers and trained peer leaders and that communications are monitored. As we state on the Facebook group itself:

The main goal of the network…is intended to be a source of support, friendship and information. It will also be used to find out what research is important to patients like you to help guide diabetes research in Canada.…As we have funding from CIHR and Diabetes Canada and have health professionals and researchers on board who will help make living with T1D easier, and will be there to answer your questions!

### Objectives

The goals of this analysis stem from T1DM-VPN’s goals of facilitating social support and identifying the priorities of young Canadians with T1DM. This protocol is for an objectives-based program evaluation, a type of evaluation that ascertains that the intended activities are taking place [14].

Our primary analysis objective is to determine whether peer leaders are (1) more central and (2) provide more social support to the group than regular members. Through analyzing group members’ exchanges, we also aim to achieve secondary objectives of (1) determining the existence and volume of 5 categories of social support and (2) identifying the issues for which informational support is requested.

Assessing the role of peer leaders has implications on the sustainability of T1DM-VPN as a program; it will help determine whether peer leaders should continue to be engaged by our team of clinicians and researchers or if regular members can rise to become “natural” peer leaders.

### Previous Analyses of Web Platforms for Youths With T1DM

Researchers have previously performed content analysis on messages authored by youths with T1DM in an online context. One study amalgamated content from 8 public T1DM forums and used an inductive coding process to categorize messages. Among the 6 resulting codes were social support, factual information, and management. The forums were not age specific, but they extracted data for which the user self-reported an age between 11 and 19 years old [15]. One research group designed an app specifically for their research study, holding regularly scheduled virtual text chats with T1DM youths aged 12 to 18 years recruited from a diabetes care center in Italy [16]. Chats were actively moderated by researchers and health professionals. This study used the same social support framework that we intend to use for coding. However, the nature of T1DM-VPN bridges that of the online platforms featured in the 2 aforementioned studies—as a private Facebook group, T1DM-VPN users are enabled the spontaneity and connectivity of a public forum but offered the privacy and peer-specific audience of the chats. T1DM-VPN users may express themselves differently because they are aware that their peers, rather than our administrative team, take the lead in answering questions and moderating discussions. Furthermore, they are aware that the group is specific to Canada and that as a result, peers may be better able to answer questions related to health care and insurance. Thus, it will be interesting to compare the frequency of different kinds of support offered across these different platforms.

The study in Italy [16] also compared the frequency of social support categories from moderators versus participants. They determined that moderators were more likely to present informational support, and participants were more likely to provide emotional support. As in this study, we will be ascertaining the relationship between a designated moderator status (peer leader vs regular member) and social support behavior. However, the nature of T1DM-VPN differs in that the designated discussion moderators are peers (ie, fellow youths with T1DM). As detailed in the “Methods” section, we will ascertain this relationship by constructing multilinear regression models that consider additional variables such as gender.

Shah and colleagues [17] have previously performed social network analysis (SNA) on youths with T1DM and their parents. They mapped participants’ subjective network of friends and relatives through interview and found that youths with a greater number of network members providing support reported lower anxiety. Saylor and colleagues [18] performed a descriptive correlational study that found that T1DM youths who were members of a student-led diabetes student organization were less likely to report poor mental health related to their diabetes than youths who were not [18]. We are able to similarly capture the network positions and behaviors of youths with T1DM who actively engage with the support network (T1DM-VPN) versus those who do not. However, T1DM-VPN differs in that it provides a virtual network of support to members, and to our knowledge, SNA has not been applied to virtual networks of T1DM patients. Moreover, our outcome of interest is not a self-reported health status, but a measure of importance in the network and the act of offering social support.

Overall, we are modelling T1DM-VPN as a social network for the purpose of understanding the role of different member types on this virtual platform. The interactions on a Facebook group may not reflect those that would occur between individuals outside a virtual network in their in-person social groups and settings.

## Methods

### Overview

Baseline information is collected upon group entry, including gender (derived from nominal information), age at diagnosis, and geographic classification (ie, large, medium, or small population centers or rural). We will manually extract interaction data from the T1DM-VPN Facebook group feed for the period between June 21, 2017 (addition of first member), and March 1, 2020. Enrollment into these groups is controlled by the researchers, and members are aware of their role in overseeing group activity. It is essential to include all members in an SNA. While we will not seek individual-level consent, we will provide a reader-friendly description of the protocol in the Facebook group and invite members to comment, as recommended by our peer leaders during a teleconference. All data will be deidentified prior to analysis by replacing names with identification numbers; the document linking these numbers to individuals will only be available to members of the research team. This proposed analysis received ethics approval from the McGill University Health Centre Research Ethics Board on May 20, 2020.

### Phase I: Directed Content Analysis

We will apply content analysis, interpreting the text and coding it into categories and then using the frequency of a category as a proxy for its importance [19]. Our content analysis will be directed, applying prior knowledge to inform coding categories. Specifically, we will apply Cutrona and Suhr’s typology [20], which is used for online support group analysis by other researchers [21,22]. Its 2 overarching categories are seeking support and providing support. These in turn each have 5 subcategories: informational, emotional, esteem, network, and tangible assistance. In addition, we will code and count social support facilitators (expressions of gratitude) as well as nonsocial support exchanges (eg, administrative messages). These two frameworks — one for social support exchanges, and another for facilitators and nonsocial support exchanges — will be adapted from Gaysynsky et al’s [22] directed content analysis of a private Facebook group for young clients of an HIV program. After applying these initial codes to the data, any content deemed not adequately coded will be further examined and discussed to determine if new categories need to be added.

Two coders will review examples of how the frameworks have been applied in Gaysynsky and colleagues’ study. First, a sample of data will be coded, analyzed for concordance between the 2 coders, and discussed to refine the consistency with which codes are applied. Then, all posts and comments will be reviewed to identify which code is most applicable. For a given comment, context (original post, preceding and proceeding comments) will be used to help inform coding decisions. A maximum of 2 codes may be applied to the same content. We will further examine the issues for which informational support is requested.

### Phase 2: Social Network Analysis

#### Defining an Interaction

In T1DM-VPN, members interact via posts and comments. Members may additionally like or react to a post or comment or vote on a poll (Table 1). Following the examples of previous SNA on Facebook [23], we will define each of these as an interaction type. We will further categorize each interaction type into a high, medium, or low engagement level (Table 1). We will also take note of who has seen a post and categorize this as a low engagement level.

**Table 1:** Types of interactions.

Engagement level and interaction type		Definition
**High**		
	Post	Content posted by any member to the group feed
	Comment	When a member comments on a post
**Medium**		
	Like/react to a post	When a member likes/reacts to a post
	Like/react to a comment	When a member likes/reacts to a comment
	Vote in a poll	When a member votes in a poll (type of post)
**Low**		
	Seen	When a member has seen a post (with no further interaction)

#### Visualizing Interactions

**Figure 2:**
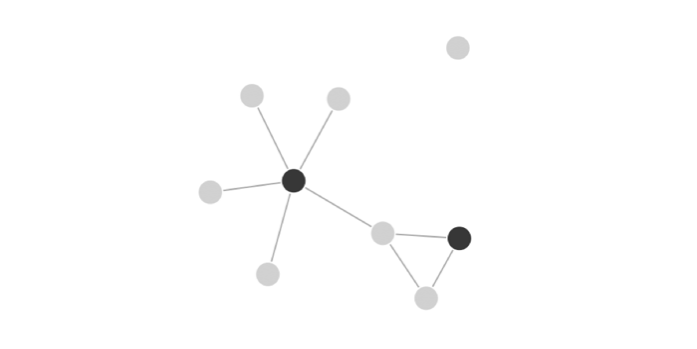
Sample visualization of a social network with 9 members. Regular members are indicated as light circles, and peer leaders are indicated as dark circles. Interactions are represented by straight, connective lines.

Based on interaction data, the social network will be visualized using programming packages specific to social network analysis, such as sna and network (R version 3.5.1 and RStudio version 1.1.456, respectively). All members (or “nodes”) will be represented as dots, and any medium or high engagement level interaction between 2 nodes will be represented with a line connecting them (Figure 2, sample visualization).

For overall network visualization, each line between nodes will represent one or more interactions for the period of interest (unweighted approach) rather than a separate line for each interaction (weighted approach). Similarly, the directionality of an interaction/line will not be indicated (undirected graph). This will render the visual representation less crowded and more interpretable. We will visualize the network both with and without peer leaders to visually appreciate their importance in driving group activity, following the example of a previous SNA evaluating the importance of a Facebook page moderator [23]. When calculating network centrality, as discussed below, we will consider both weighted and unweighted approaches.

#### Network Dynamics and Time in SNA

We will evaluate network dynamics over approximately a 3-year period. In epidemiological terms, this is an open cohort, in that members may leave or join at different times. In prior Facebook group studies, researchers have taken a data sample from what they assumed to be a stable time frame. They then take absolute (eg, total number of posts) or per-person (eg, average number of posts per person) measurements [22,24].

Instead of making assumptions about the stability of a network, we will actually identify stable time frames by modeling monthly membership growth as a Poisson process, a process in which the average rate of events is known but the exact timing of events is random. We propose that any month for which the probability of observed growth falls below 50% (ie, is statistically unusual based on the known average rate of events) represents the end of a stable time interval and the start of a new stable interval. For several network and node attributes detailed below, we will calculate an incident measure for each time interval, then a summary measure across all intervals if applicable. These calculations are detailed below.

#### Network Attributes

Social networks can be characterized by measures of density, identification of cliques, and indicators of engagement [25]. Density is the total number of interactions divided by the total possible number of interactions. A clique is a subgroup of nodes that are directly connected to one another, with no node being connected to all in the subgroup. Quantifying engagement typically involves an enumeration of high engagement level interactions; we will use number of posts per person-month. These 3 attributes will be calculated for each time interval. To summarize them, the difference in density and number of cliques from the first interval to the last will be calculated, and the mean number of posts per person-month will be taken.

#### Node Attributes

Just as the network may be characterized by defined metrics, nodes may also be characterized in terms of their centrality and in terms of the nature of their interactions. For each node, we will calculate the proportion of all their interactions that are categorized as high, medium, or low engagement level (Table 1). This helps identify members who actively participate in discussion versus those who view others’ interactions but infrequently participate themselves. We will also calculate the proportion of a user’s interactions that are categorized as offering social support; this is one of the measures that will be used in the regression analysis. Because these are proportions, there is no need to additionally account for group stability.

Centrality is the other measure that will be used in the regression analysis, but it requires accounting for stability. Centrality refers to one’s prominence in the network. In virtual SNA, prominence is calculated using virtual connections. In calculating degree centrality specifically [25,26], researchers may consider the total number of nodes with which a node is connected or the total number of connections, including repeated connections with the same node. We have developed a measure that incorporates both of these aspects. In ascertaining centrality, we will focus only on medium and high engagement level interactions as connections. In order to capture both breadth and depth of connection in centrality, we have adapted modeling methods from economics [27,28], creating a measure that is a convex combination of the weighted and unweighted approaches:

Centrality = α × total No. of connections + (1 – α) × total No. of interactions

In the above equation, we choose α to be .8. Although α is usually any number between 0 and 1, we will be testing values between .5 and 1 in a sensitivity analysis. This range ensures that breadth of connections is given equal or greater weight than depth.

The centrality of each member will be calculated for each stable time interval (ie, include interactions occurring exclusively within that interval), then standardized to that of the most central member for that interval. The resulting measures for each member will be averaged. This final mean value will be used in regression analysis.

Rather than taking a direct calculation of these attributes, as existing virtual SNAs have, we are adopting this approach because we appreciate that length of membership in T1DM-VPN is similar to exposure time. If a user has been involved in the group for a longer period of time, then their increased centrality may be a function of their increased opportunity to interact with others. We also understand that the number of possible connections exposed to a user at a given point in time differs. Network membership increases over time, and so does the number of potential connections for a user. By taking measures of centrality from smaller, stable time frames, we can better control for these two potential confounders.

### Phase 3: Regression Analyses

We will evaluate the relationship between network centrality and designated peer leader status to determine whether those so designated are more likely to be central within the network. We will apply multivariate linear regression, considering inclusion of the following variables in the model: gender, age at diagnosis, and geographic classification (ie, large, medium, or small population centers or rural). Studentized residuals will be used to detect potential outliers. We will then calculate a Bayesian information criterion (BIC) for each model, where a lower BIC indicates a better model. We will also use multiple linear regression to assess whether peer leader status is correlated with the individual’s proportion of interactions offering support.

## Results

T1DM-VPN was launched in June 2017, and as of March 1, 2020, it has 196 patient-members. This research protocol received ethics approval from the McGill University Health Centre Research Ethics Board on May 20, 2020. Baseline information about each group member was collected upon their addition into the group. Collection of interaction data is set to be complete by fall 2020, and data analysis is set to be conducted by November 2020.

## Discussion

### Contribution to the Literature

Peer-led virtual networks with some professional oversight and access are promising avenues to enhance chronic disease management in general and diabetes self-management in particular. To properly understand their mechanics and impacts, traditional methods in epidemiology and social network analysis need to be adapted and applied. The proposed analytic approaches aim to do this. It will be interesting to interpret our results in the context of previous analyses of T1DM support networks that differ slightly in geographic scope, target population, and platform function. Furthermore, to our knowledge, this will be the first analysis of a virtual T1DM patient community of this level of comprehensiveness. The findings may be of interest to professionals aiming to launch a similar virtual community in another population, country, or region.

Zhou and colleagues [29] have proposed a conceptual framework for social media–based health information management. Though commonly applied to professional-patient information exchange, it may also be used to understand peer-peer interactions; health information management refers broadly to the activities that users perform in order to process health information items to fulfill their needs. This includes needs for social support. The social media–based health information management framework outlines processes by which users aim to improve 4 outcomes, represented as the “4Cs”: convenience, care, communication efficiency, and cost-effectiveness.

In their framework, Zhou and colleagues [29] focus on information processing as performed by researchers on social media data generated by users. However, we also find it useful to conceptualize T1DM-VPN members as engaging in the processes of generating and retrieving health information amongst themselves, as well as integrating and applying it in their personal lives. As a social media platform, T1DM-VPN may improve the convenience with which young Canadians living with T1DM exchange information with one another. The other *C*’s are currently less pertinent to T1DM-VPN.

Our aim is to understand if T1DM-VPN is providing support and stimulating engagement because this will inform how we will approach sustainability avenues. Thus far, our research team has actively invested time and resources to train the national panel of peer leaders and sustain their participation. Our team also recruits new T1DM-VPN members and moderates the community.

While growth of the network itself is indeed important, through the proposed analysis we will determine the degree of interaction within the network and the types of support offered. Demonstration of a high level of engagement and support would provide a strong rationale to develop a sustainability strategy. Another goal of our analyses is to determine whether central roles remain the purview of the selected peer leaders or whether regular members are naturally taking on the role of peer leaders (ie, posting often, inviting new members, offering social support). If that is the case, then there may be less need for us to recruit leaders and provide them with a stipend. The ultimate goal is for T1DM-VPN to become at least a partially self-sustaining group, with our research team transitioning from less of an active role to more of an administrative role.

We believe that our study will build evidence and provide a road map for the building and maintenance of virtual peer support networks in chronic disease.

### Knowledge Dissemination

We will promote and disseminate our approach and findings via scientific manuscripts. We will also share findings through the Diabetes Canada website, social media, and professional conferences. In our consultation with the peer leaders to develop this analysis protocol, they expressed great interest in seeing for themselves how T1DM-VPN has developed over the years. Thus, in keeping with our participatory approach, we will share and discuss our results directly with our peer leaders. Findings may also be presented to T1DM attendees of patient conferences, such as No Limits with T1D: Inspire, Empower, Connect, which is held annually in Vancouver, British Columbia, Canada.

## Abbreviations

BIC: Bayesian information criterion
CIHR: Canadian Institutes of Health Research
DIY: do-it-yourself
HbA_1C_: glycated hemoglobin
MUHC: McGill University Health Centre
SNA: social network analysis
T1DM: type 1 diabetes mellitus
T1DM-VPN: Type 1 Diabetes Mellitus Virtual Patient Network
